# Should all elective knee radiographs requested by general practitioners be performed weight-bearing?

**DOI:** 10.1186/2193-1801-3-707

**Published:** 2014-12-02

**Authors:** Alvin Chen, Joshua Balogun-Lynch, Kavita Aggarwal, Elizabeth Dick, Chinmay M Gupte

**Affiliations:** Mr Alvin Chen, Specialist Registrar Trauma & Orthopaedics, Royal National Orthopaedic Hospital, Stanmore, Middlesex, HA7 4LP UK; Dr Joshua Balogun-Lynch, Foundation Year 1, Northwick Park Hospital, Harrow, HA1 3UJ UK; Dr Kavita Aggarwal, Foundation Year 1, East Surrey Hospital, Redhill, Surrey, RH1 5RH UK; Dr Elizabeth Dick, Consultant Radiologist, St Mary’s Hospital, Imperial College NHS Trust, Praed Street, London, W2 1NY UK; Mr Chinmay Gupte, Consultant Orthopaedic Surgeon/Senior Lecturer, St Mary’s Hospital, Imperial College NHS Trust, Praed Street, London, W2 1NY UK

**Keywords:** Weight bearing, Knee radiograph, Knee osteoarthritis

## Abstract

The aims of this study were to: [1] Assess the number of patients with suspected knee osteoarthritis that underwent repeat weight-bearing(WB) knee radiographs in the orthopaedic clinic following initial non-WB radiograph requested by their general practitioner (GP). [2] Confirm whether repeating WB knee views changed radiology reports. [3] Determine the number of London trusts with protocols for routinely performing WB views. A Retrospective cohort study of 1968 patients aged >40 years referred to a London teaching hospital for knee radiographs over 12 months. Radiographs were identified as WB/non-WB. Subsequent repeat WB views performed in those that went on to have an orthopaedic consultation were also documented. A consultant musculoskeletal radiologist reported both images. A proforma containing a likert scale of severity for commonly reported abnormalities in knee osteoarthritis and criteria from the Kellgren and Lawrence scale was used for reporting. London NHS Trusts were surveyed to identify if protocols were in place for performing WB views. A total of 1,968 patients underwent knee radiographs, of which 1922 (97.7%) had initial non-WB radiographs. Of the 56 patients in this group that underwent required repeat WB radiographs, joint space narrowing was reported as more severe on WB versus non-WB radiographs (p = 0.035). Only 54% of departments routinely performed WB radiographs. Few patients (2.3%) referred by GPs have WB radiographs requested. Some of those referred for a specialist opinion required repeat WB views. Nearly half of London hospitals do not routinely perform WB radiographs. This represents a significant financial burden to the NHS, increased radiation exposure and wasted patient/clinician time. We propose that all GP requested knee radiographs be performed as WB unless otherwise stated.

**Electronic supplementary material:**

The online version of this article (doi:10.1186/2193-1801-3-707) contains supplementary material, which is available to authorized users.

## Introduction

Osteoarthritis represents a complex musculoskeletal disorder with multiple genetic, constitutional and biomechanical risk factors. It represents the most common form of joint disease in the elderly and ranks amongst the top 5 causes of disability (Murray and Lopez 1997). The prevalence of knee osteoarthritis in patients aged over 45 in the general practice (GP) setting has been estimated at 12.5% in 2005 (Bedson et al. 2005).

The diagnosis of knee osteoarthritis is based on clinical history and examination, followed by radiographic examination of the knee (Altman et al. 1986). Radiographs can be taken with the patient supine or standing. The Kellgren-Lawrence Grading Scale is one measure used to assess knee osteoarthritis severity on plain radiograph (Kellgren and Lawrence 1957). Studies have shown that standing/weight-bearing (WB) views improve the detection of joint space narrowing more reliably than supine views (Brandt et al. 1991). This is probably because WB places compressive stresses on the knee joint that help accentuate any loss of articular cartilage causing joint space narrowing. It is not known whether knee radiographs requested by the general practitioner are routinely performed WB or non-WB in UK hospitals.

Professor Briggs highlighted in his report ‘*Getting it right first time’* that 15%-30% of GP consultations will be of a musculoskeletal nature (Briggs 2012). With regard to requesting musculoskeletal investigations, in a survey of GPs in 2003 by Bedson et al. (Bedson et al. 2003), 50% of GPs were confident of diagnosing osteoarthritis on the basis of the plain radiograph. Furthermore, 80% of GPs were likely to request a radiograph if considering referral to an orthopaedic surgeon. The radiological report of the GP-requested knee radiograph is therefore pivotal in determining whether onward specialist referral is undertaken. The radiology report is however, influenced by whether WB or non-WB views are taken (Brandt et al. 1991).

The Royal College of Radiologists has published guidelines on the indications for various imaging investigations, including plain radiography of the knee. In an audit of 1153 knee radiographs requested by GPs, only 50% of those radiographs fell within the RCR guidelines (Morgan et al. 1997). Morgan et al. found in 87% of cases, there was no change in management, apart from continuation of symptomatic treatment. GPs in Morgan’s study reported medico-legal reasons as a significant factor in unnecessary radiograph requests. One further reason that management may not have changed in the case of knee radiographs, could be that non-WB views were taken, which did not fully reveal the joint space loss from degenerative change that WB views would have revealed.

### Aims

The aims of this study were to:

1. Quantify the number and cost of repeat radiographs performed for the diagnosis of osteoarthritis of the knee at our institution, a major London teaching hospital.
2. To confirm whether WB and/or skyline views significantly change the formal report by a radiologist regarding the presence of osteoarthritis, given the reliance of GPs on such reports to make the diagnosis of osteoarthritis.
3. To determine how many radiology departments in London have implemented policies on routinely performing WB views on knee radiograph requests suspecting osteoarthritis.

## Methods

### Aim 1

All patients over the age of 40, undergoing radiographic imaging of the knee at our institution were included in this retrospective cohort study. The original data set included only non-trauma patients. Patients with any previous fixation or arthroplasty involving the knee, duplicate entries, no image available to view, no WB label on image or Rosenberg views were excluded. Knee radiographs taken in full extension between 1st January 2011 to 31st December 2011 were included in the study. It was not the policy of the radiology department at our institution, at the time of the study, to routinely perform WB or skyline views of the knee unless specifically requested to do so by the clinician.

Radiographs were examined to identify if they were WB or non-WB. All patients who then had subsequent WB knee radiographs after an orthopaedic consultation in the following 12 months were identified.

The cost of these repeat procedures was calculated based on financial information provided by the radiology department. The radiation dose was calculated utilizing a sample of patients to find the average radiation dose in millisieverts (mSv) patients’ received whilst having a knee radiograph.

### Aim 2

All patients who initially had extended non-WB radiographs of the knee, then subsequently had repeat extended radiographs with WB and/or skyline views, were noted. The radiographs of these patients were then reported by a consultant radiologist at our institution. The consultant radiologist was asked to grade the severity of each radiographic feature between 1 (least severe) and 5 (most severe). A proforma containing a list of the commonly reported abnormalities in radiology reports of the knee from Bedson et al. 2007, was used by the consultant as a template for the reporting (see Additional file 1). This also included criteria from the Kellgren-Lawrence Grading Scale for knee osteoarthritis (1957). Results of non-WB vs WB radiographs of the knee were analysed.

Statistical analysis was performed using SPSS (version 20). As the data was discrete and non parametric a chi-squared test for trend was employed to analyse for significance with a p < 0.05.

### Aim 3

A telephone survey of 35 acute NHS hospitals in the London region was conducted during a two-week period. The list of these trust was obtained from the NHS choices website (NHS 2013). The superintendent radiographer of each hospital was asked if their department had a protocol for routinely performing WB anterior-posterior (AP), and lateral radiographs of the knee suspecting osteoarthritis. Details of the protocols for each department contacted were recorded.

## Results

### Aim 1: radiographs performed for GPs & repeats

Of the initial 2,086 patients who had knee radiographs requested their GP, 118 were excluded due to: previous arthroplasty (43), duplicate entries (27), no image available to view (24), no WB label on image (23), Rosenberg view (1). This left 1,968 patients to be included in the analysis. The mean age of the patients was 61.7 years (SD 12.5).

Of the 1968 patients, 2.3% (n = 45) of patients had their initial radiograph specifically requested by their GP to be performed as a WB radiograph.

2.9% (n = 56) of patients had an initial GP requested non-WB radiograph, and subsequently had repeat WB radiographs performed at the request of the orthopaedic surgeon.

### Aim 2: radiologist reporting

**Figure 1:**
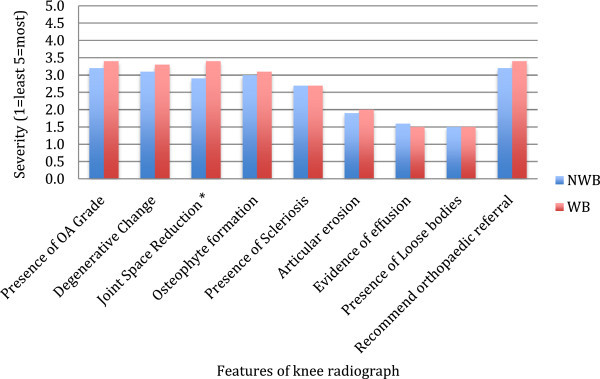
**Graph to show the comparison between WB and Non-WB radiographs on formal reporting.** *represents a p < 0.05.

Joint space narrowing was reported as more severe on the WB radiographs when compared to the non-WB radiographs, and this difference reached statistical significance (p = 0.035). All the other characteristic features seen on knee radiographs suggesting osteoarthritis showed a trend to be more severe on the WB views (except loose bodies), but these did not reach significance (p > 0.05) (See Figure 1).

### Aim 3: telephone survey results

Of the 35 radiology departments included in the survey, 19 (54%) had a protocol in place for routinely performing WB AP radiographs on patients. Within this group, 2 departments (6%) would only perform WB AP views if the request form indicated suspected osteoarthritis. One department had an age dependent policy, whereby only patients over 30 would have WB AP views performed routinely. Departments with no protocol in place would only perform WB AP radiographs on patients if a WB view had been specifically requested by the GP. None of the departments performed skyline views routinely.

### Cost

The cost of repeating a second radiograph (AP and Lateral view only) was calculated at £22. As 56 repeat radiographs were performed during Jan 2011-December 2011, this equates to an extra £1232 added cost to the department. The time cost to the radiographer over this period was calculated to be 9 hours 20 minutes (based a mean of 10 minutes for each extra set of radiographs).

The quoted radiation dose for a standard chest radiograph is 0.02 mSv (Public Health England 2014). The average extra radiation dose received by a patient having an AP/Lateral film was calculated to be 0.0107 mSv, approximately half that of a standard chest radiograph.

Applied across all London hospitals the added cost of repeat radiographs in the 16 London hospitals without WB protocols would be £19,712. Extrapolating this to 46% of the 168 NHS trusts in the UK, this would equate to an extra unnecessary direct cost to the NHS of £95,208 per annum. Extrapolating this to include the 353 NHS hospitals in England, this cost would be £200,052.

## Discussion

This study shows that, in the absence of a departmental policy for routinely performing WB knee radiographs, the vast majority of non-traumatic knee radiographs requested by GPs in patients over the age of 40 are performed non-WB (98%). In the financial year of 2011 – 2012 the national cost of all diagnostic radiology to the NHS budget was £815 million (Department of Health 2012). To put the extrapolated unnecessary direct costs (£200,052) calculated from our study into context this would account for 0.025% of diagnostic radiology costs. In the current politico-economic climate, this saving, while modest compared to the whole NHS budget, represents a significant and unnecessary cost, which can be readily and easily addressed.

Our department sees approximately 1000 new knee patients annually. Of these patients, approximately 300 patients (in 2012) would fit our inclusion criteria (age over 40, no previous arthroplasty or fixation, no trauma). Therefore, as a representation of patients seen by our department for suspected knee osteoarthritis, 19% (56 out of 300) require an unnecessary repeat of the knee imaging at the time of their outpatient visit. This would therefore support the view that a significant amount of time is wasted in the outpatient setting repeating these radiographs. The overall numbers of patients actually requiring such repeat radiographs remain, however relatively small (n = 56). However, this may be due to the reluctance of specialist clinicians in subjecting patients to further radiation in repeating radiographs as WB.

There is good evidence that tibio-femoral joint space narrowing is good evidence for cartilage loss (Buckland-Wright et al. 1995). In addition, our study confirms that WB compared to non-WB films significantly increase the amount of joint space narrowing on plain radiographs (p = 0.035), and hence the severity of osteoarthritis when reported by a radiologist. This represents an area of possible delayed or missed diagnosis, with its potential costs in terms of complaints and/or litigation.

From the results of our study, the numbers of patients who may be suffering from such a delay could potentially be quite large, with a pool of up to 1867 patients (those who had non-WB radiographs and did not have repeat WB radiographs) being under-diagnosed with regard to the extent of their knee osteoarthritis. This may then result in delayed/inappropriate treatment or referral of such patients to specialist care.

Jayatilaka et al. (2012) in a study of 41 patients over a two-week period found none of the patients referred by GPs to orthopaedic outpatients had WB radiographs requested prior to consultation. A higher proportion of patients in our study had WB knee radiographs requested prior to being seen in our orthopaedic clinic (2.3%), however a significantly lower amount of radiographs initially performed non-WB were repeated in our study (2.9% vs. 25%). In our literature search this was the only study found looking at the requesting trends of GPs with regards to WB and non-WB knee radiographs. Given the time period and sample size of our study, we believe our findings to be a comprehensive representation of plain radiograph requests by GPs investigating knee osteoarthritis.

Our study raises the question whether a national policy to routinely perform elective knee radiographs with the patient WB should be implemented, or at the very least discussed between primary care institutions, radiology and orthopaedic departments. In the absence of such a national policy existing, our study suggests that it would be prudent for doctors in all specialities to request all AP and lateral knee radiographs as weight bearing (or “erect”) views, unless there is a history of trauma or the patient is not able to bear weight on the knee.

### Limitations of the study and areas for future research

A limitation of our study is that patients who had an initial radiograph at our institution but subsequently referred by their GP to another institution for their knee pathology would not be included in our results. Likewise, patients who have had their initial radiograph elsewhere and then subsequently referred to the orthopaedic department at our institution would not have their imaging available to view on our system. However, we believe these numbers to be relatively small.

In addition, the reporting radiologist was not blinded as to whether the radiographs were WB or non-WB, thus potentially biasing reporting. However we regard joint space loss as a significantly objective measure of degeneration for bias to have a minimal influence. Further studies on WB vs. non-WB views should however involve reporting by a radiologist who is blinded to the manner in which the films were taken. The WB and non-WB images were each viewed once by a single radiologist in this study when reporting therefore intra-observer and inter-observer variation was not calculated. Further studies should involve more than one blinded radiologist to report images more than once in order for this to be determined.

Our study has investigated the direct costs of repeating radiographs in patients, but there also remain additional un-quantified indirect costs, which include, wasted clinical time - a service cost - to the clinicians and radiographers who might otherwise be able to use that time treating other patients. Other indirect costs include the potential of delayed or missed diagnosis of osteoarthritis, further unnecessary investigations ordered such as MRI, unnecessary radiation exposure to the patient and also causing, in general, a poorer patient experience, a subject of increasing relevance in these times.

## Conclusion

Only a small proportion of patients referred for knee radiographs by GPs have WB AP films. The radiographic features of osteoarthritis appear to be more severe on WB plain radiographs of the knee when compared with non-WB plain radiographs of the knee. In London, 46% of hospitals do not routinely perform WB radiographs to investigate suspected knee osteoarthritis, potentially leading to a delay in diagnosis, referral or treatment of these patients. It represents a significant risk and cost burden to the NHS. In the absence of a national policy on WB status on knee radiographs, we would recommend when investigating for knee osteoarthritis, radiographs be requested at “weight bearing” or “erect” when general practitioners complete the request form as part of a national policy.

## Electronic supplementary material

Additional file 1: **PROFORMA for Reporting.** (DOCX 40 KB)

## Abbreviations

GP: General practitioner
WB: Weight bearing
NHS: National health service
MSV: Millisieverts
AP: Anterio-posterior.
