# Supplementary material for: Should all elective knee radiographs requested by general practitioners be performed weight-bearing?
Source: Springerplus. 2014 Dec 2;3:707. doi: 10.1186/2193-1801-3-707 (PMC4265640; doi:10.1186/2193-1801-3-707)
Supplement: Supplementary file 1 — Additional file 1: PROFORMA for Reporting. (DOCX 40 KB) [file 40064_2014_1409_MOESM1_ESM.docx]

**Supplementary material:**

**PROFORMA for Reporting**

**DOB: Patient Initial: XR1: XR2:**

**A ‘Normal’ X-ray: 1=not likely – 5=very likely**

1 2 3 4 5

***Please answer the following questions as:***

**1: None / 2: mild / 3: moderate / 4:severe / 5:very severe**

Presence of OA Grade –

1 2 3 4 5

Degenerative Change

1 2 3 4 5

Joint space reduction

1 2 3 4 5

Osteophyte formation

1 2 3 4 5

Presence of sclerosis

1 2 3 4 5

Articular erosion

1 2 3 4 5

Radiological presence of effusion

1 2 3 4 5

Presence of loose bodies

1 2 3 4 5

***Please answer the following question as:***

**1=not likely – 5=very likely**

Likelihood of recommendation for orthopaedic referral:

1 2 3 4 5
